# Real-time and simultaneous monitoring of the phosphorylation and enhanced interaction of p53 and XPC acidic domains with the TFIIH p62 subunit

**DOI:** 10.1038/oncsis.2015.13

**Published:** 2015-06-01

**Authors:** M Okuda, Y Nishimura

**Affiliations:** 1 Graduate School of Medical Life Science, Yokohama City University, Yokohama, Japan

## Abstract

Posttranslational modifications have critical roles in diverse biological processes through interactions. Tumor-suppressor protein p53 and nucleotide excision repair factor XPC each contain an acidic region, termed the acidic transactivation domain (TAD) and acidic fragment (AF), respectively, that binds to the pleckstrin homology (PH) domain of the p62 subunit of the transcription factor TFIIH. Human p53-TAD contains seven serine and two threonine residues, all of which can be phosphorylated. Similarly, XPC-AF contains six serine and two threonine residues, of which Thr117, Ser122 and Ser129 have been reported as phosphorylation sites *in vivo*, although their phosphorylation roles are unknown. Phosphorylation of Ser46 and Thr55 of p53-TAD increases its binding ability; however, the role of XPC-AF phosphorylation remains elusive. Here we describe a system for real-time and simultaneous monitoring of the phosphorylation and p62-PH affinity of p53-TAD and XPC-AF using nuclear magnetic resonance (NMR) spectroscopy. Unexpectedly, among seven reported kinases that presumably phosphorylate Ser46 and/or Thr55 of p53-TAD, only two specific and high-efficiency enzymes were identified: JNK2α2 for Ser46 and GRK5 for Thr55. During interaction with p62-PH, four different affinity complexes resulting from various phosphorylation states of p53-TAD by the kinases were identified. The kinetics of the site-specific phosphorylation reaction of p53-TAD and its affinity for p62-PH were monitored in real-time using the NMR system. Isothermic calorimetry showed that phosphorylation of Ser129 of XPC-AF increases binding to p62-PH. Although CK2 was predicted to phosphorylate Ser122, Ser129 and Ser140 from its sequence context, it specifically and efficiently phosphorylated only Ser129. Simultaneous monitoring of the phosphorylation and augmentation in p62-PH binding identified a key residue of p62-PH for contacting phosphorylated Ser129. In summary, we have established an NMR system for real-time and simultaneous monitoring of site-specific phosphorylation and enhancement of affinity between phosphorylation domains and their target. The system is also applicable to other posttranslational modifications.

## Introduction

The tumor-suppressor p53 has critical roles in DNA repair, cell cycle arrest, apoptosis, senescence, metabolism and autophagy in response to cellular stress.^1, 2, 3, 4^ One of the reasons why p53 can be an exceptionally multifunctional protein is its ability to fulfill paradoxical interactions, in other words, it has specific and malleable interactions with various partners. The acidic transactivation domain (TAD; residues 1–61) at the N-terminus is important for p53 transcription activity through its interaction with transcriptional co-activators, co-repressors and general transcription factors. p53-TAD consists of two homologous subdomains, TAD1 (residues 1–42) and TAD2 (residues 43–61). Both domains function as hubs, contacting multiple partners in intricate networks.

p53 is subjected to diverse posttranslational modifications, which contribute significantly to its specific and malleable interactions. In particular, p53-TAD is subject to phosphorylation.^5, 6^ Human p53-TAD has nine phosphorylation sites: Ser6, Ser9, Ser15, Ser20, Ser33, Ser37, Ser46, Thr18, and Thr55, all of which have been implicated in activation of p53 upon stress.^7^ For example, phosphorylation of Ser46 activates p53 apoptotic activity,^8, 9^ whereas phosphorylation of Thr55 is involved both in G1 cell cycle progression via the degradation of p53,^10^ and in nuclear export of p53.^11^

As demonstrated for p53 and many key hub proteins, phosphorylation and other posttranslational modifications are essential for modulating intermolecular interactions. For instance, single-site and multiple-site phosphorylation of Ser15, Thr18, Ser20, Ser33, Ser37, Ser46, and Thr55 in human p53-TAD increase the affinity of p53 for the TAZ1/CH1, KIX, TAZ2/CH3 and NCBD/IBiD domains of its co-activators, CBP and p300.^12, 13, 14, 15^ In addition, phosphorylation of Thr18 decreases the interaction of p53 with its main negative regulator HDM2/MDM2,^16^ and phosphorylation of Ser46 and Thr55 increases its p53 affinity for the pleckstrin homology (PH) domain of the p62 subunit of the general transcription factor TFIIH.^17, 18^ p62 is also a critical hub protein, functioning in both transcription and DNA repair.

Similar to p53-TAD, the acidic fragment (AF; residues 109–156) of the nucleotide excision repair factor XPC binds to p62-PH.^19, 20, 21^ XPC-AF contains six serine and two threonine residues, of which Thr117, Ser122 and Ser129 have been reported as phosphorylation sites *in vivo*,^22, 23^ although their phosphorylation roles are unknown.

A large number of studies have demonstrated that, in response to stress, p53 is stabilized and activated by altering its interactions with its binding partners via phosphorylation and other posttranslational modifications. However, few methods for monitoring its changing interactions in real time upon modification at an atomic level have been described thus far. To address this issue, here we have used real-time nuclear magnetic resonance (NMR) spectroscopy^24^ to study the interaction of the p53-TAD, as well as XPC-AF, with p62-PH. First, we identified highly specific kinases for p53-TAD, namely c-Jun NH2-terminal kinase 2α2 (JNK2α2) for Ser46 and G protein-coupled receptor kinase 5 (GRK5) for Thr55, and verified the high efficiency of these kinases by analyzing the kinetics of the phosphorylation reactions. After demonstrating the existence of four different affinity complexes of p53-TAD and p62-PH arising from the different phosphorylation states of p53-TAD, we simultaneously monitored the site-specific phosphorylation and augmenting interaction of p53-TAD with p62-PH in real time. For XPC-AF, we first showed that Ser129 was specifically and efficiently phosphorylated by casein kinase II (CK2), which strengthened binding of XPC-AF to p62-PH. We then monitored the enhancement in complex formation mediated by Ser129 phosphorylation in real time by NMR, identifying a key residue of p62-PH for interaction with phosphorylated Ser129 of XPC-AF.

## Results

### JNK2α2 and GRK5 specifically phosphorylate Ser46 and Thr55 of p53-TAD, respectively

**Figure 1:**
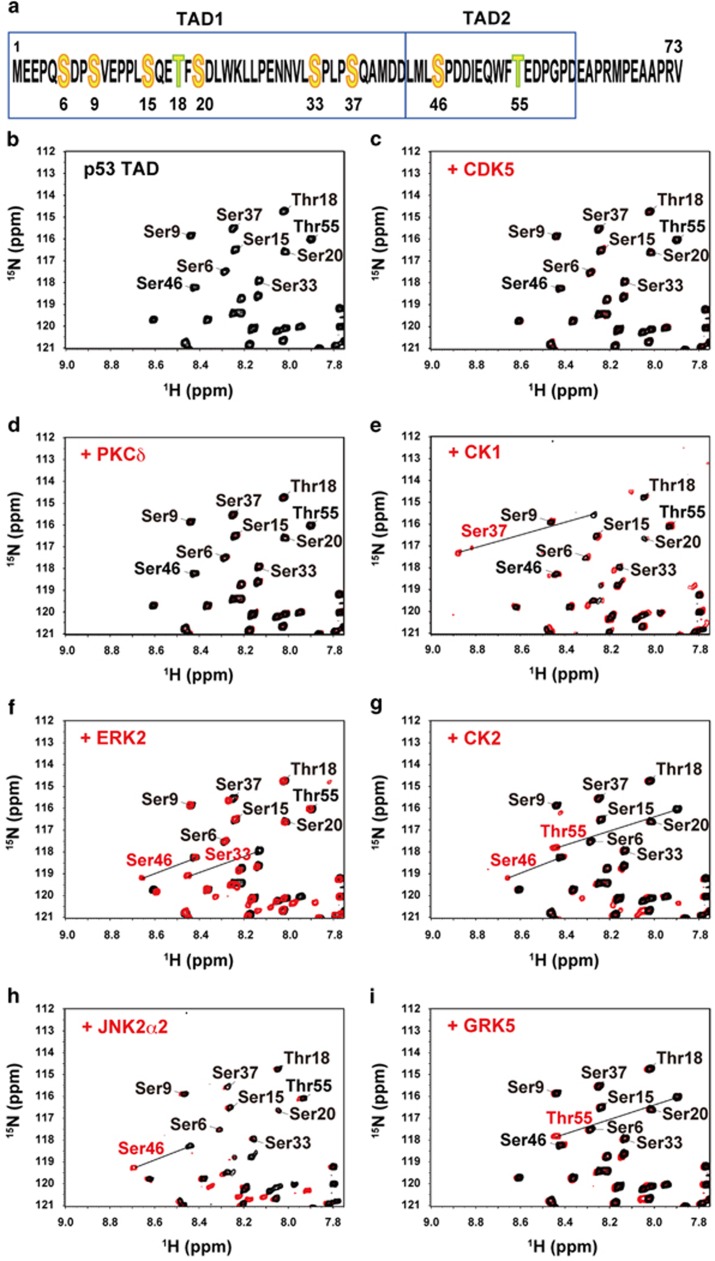
Screening for kinases that specifically phosphorylate Ser46 or Thr55 in p53-TAD. (**a**) Amino-acid sequence of p53-TAD. (**b**) ^1^H,^15^N HSQC spectrum of p53-TAD alone. (**c**–**i**) Overlay of ^1^H,^15^N HSQC spectra of p53-TAD alone (black) and the mixture of p53-TAD with the following kinases (red): (**c**) CDK5, (**d**) PKCδ, (**e**) CK1, (**f**) ERK2, (**g**) CK2, (**h**) JNK2α2, and (**i**) GRK5.

Initially, we attempted to establish a system for the real-time and simultaneous monitoring of the dual phosphorylation of Ser46 and Thr55 located in the TAD2 region of p53 and the resulting increased interaction of p53 with p62-PH. We therefore sought to identify kinases specific for each residue of p53-TAD and tested well-known candidate kinases: cyclin-dependent kinase 5 (CDK5)^25^ and protein kinase Cδ (PKCδ)^26^ for Ser46; extracellular signal-regulated kinase 2 (ERK2)^27^ and GRK5^28^ for Thr55; and CK1, CK2 and JNK2α2, three kinases that are known to phosphorylate residues Ser20, Ser33, Ser37, Ser46, Thr18 and Thr55.^14^ We added each candidate kinase (2.5 μg) to 100 μl of 50 μM ^15^N-labeled p53-TAD (residues 1–73) (Figure 1a) and monitored whether a phosphorylation reaction occurred at Ser46 or Thr55 using NMR (Figures 1b–i).

CDK5 and PKCδ did not phosphorylate any residues under the conditions used (Figures 1c and d). We therefore independently verified the activity of both kinases for their substrate peptides by NMR: CDK5 specifically phosphorylated a histone H1 peptide (PKTPKKAKK) and PKCδ phosphorylated a PKC peptide (ERMRPRKRQGSVRRRV) (Supplementary Figure S1). CK1 specifically phosphorylated Ser37 (Figure 1e). Although ERK2 has been reported to phosphorylate Thr55, we found that it efficiently phosphorylated Ser33 and also Ser46 (Figure 1f). CK2 phosphorylated both Ser46 and Thr55; however, the reaction seemed to be comparatively slow, especially for Ser46 (Figure 1g). Overall, highly specific phosphorylation of Ser46 was obtained with JNK2α2 (Figure 1h) and that of Thr55 with GRK5 (Figure 1i).

### Kinetics of the site-specific phosphorylation reactions of JNK2α2 and GRK5

**Figure 2:**
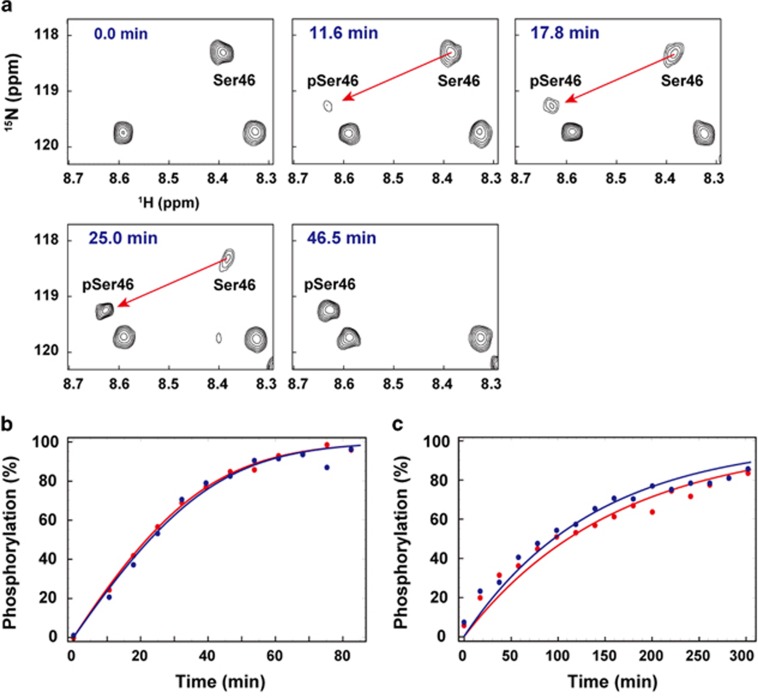
Real-time NMR monitoring of the phosphorylation of p53-TAD. (**a**) ^1^H,^15^N HSQC spectra of p53-TAD before and after the addition of JNK2α2. (**b**) Build-up curve showing time course of phosphorylation of p53-TAD at Ser46 by JNK2α2 (1.0 μg). (**c**) Build-up curve showing time course of phosphorylation of p53-TAD at Thr55 by GRK5 (2.5 μg). The curves were calculated by using data from the phosphorylated residue and its neighboring residues (Supplementary Figure S3). Each experiment was repeated twice.

To examine the kinetics of the enzymatic reactions of JNK2α2 and GRK5, phosphorylation of p53-TAD was monitored in real time by NMR (Figure 2). Using 150 μl of 25 μM ^15^N-labeled p53-TAD as a substrate, 1.0 μg of JNK2α2 achieved ~100% of phosphorylation at Ser46 within 1 h (Figures 2a and b and Supplementary Figures S2 and S3). In contrast, 2.5 μg of GRK5 took ~5 h to achieve ~80% phosphorylation of Thr55 (Figure 2c and Supplementary Figures S2 and S3).

### Four different affinity complexes result from the phosphorylation states of p53-TAD

**Figure 3:**
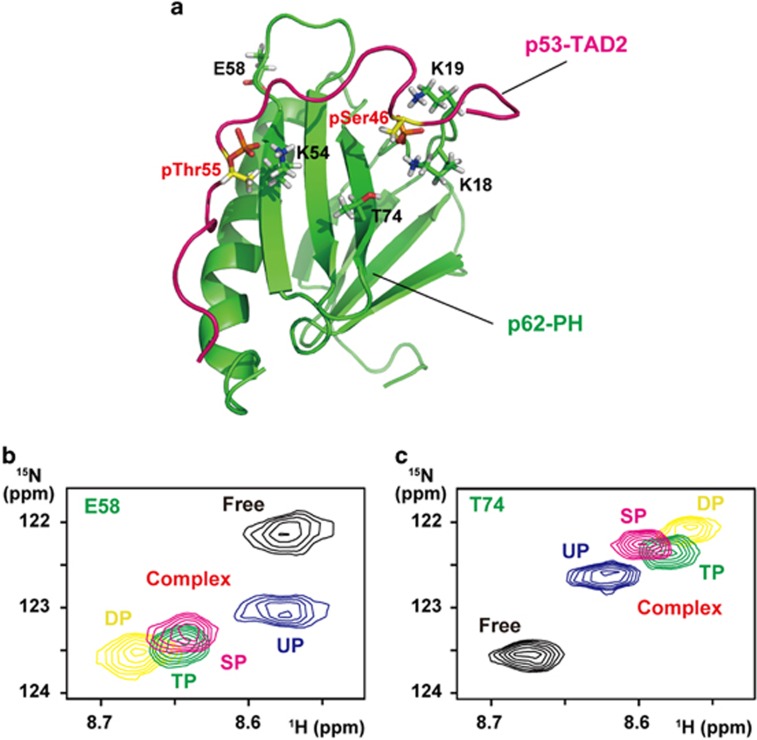
NMR signals of p62-PH bound to p53-TAD2 in different phosphorylation states. (**a**) Structure of the complex between Ser46, Thr55 double-phosphorylated p53-TAD2 and p62-PH (PDB code 2RUK). (**b** and **c**) Superposition of five ^1^H,^15^N HSQC spectra of p62-PH alone (black) and of the complex of p62-PH with unphosphorylated p53-TAD2 (UP, blue), Ser46-phosphorylated p53-TAD2 (SP, magenta), Thr55-phosphorylated p53-TAD2 (TP, green) and Ser46, Thr55 double-phosphorylated p53-TAD2 (DP, yellow) showing signals from (**b**) E58 and (**c**) T74 of p62-PH.

In a previous study, we determined the structure of the complex between the p53-TAD2 peptide (residues 41–62), doubly phosphorylated on Ser46 and Thr55, and p62-PH of TFIIH using NMR and revealed that phosphorylated Ser46 and Thr55 make electrostatic contacts with K18/K19 and K54, respectively, of p62-PH (Figure 3a).^18^ (Note that, to avoid confusion, residues of p53 and XPC are represented by three-letter code and those of p62 are represented by one-letter code in this paper.) We also quantitatively examined the effects of phosphorylation of p53-TAD2 on binding to p62-PH by using isothermal titration calorimetry (ITC) and NMR. Single phosphorylation of Ser46 or Thr55 increased the binding ~4-fold (*K*_d_=6 μM) or ~5-fold (*K*_d_=5 μM), respectively, and dual phosphorylation further enhanced the binding ~25-fold (*K*_d_=1 μM).^18^ Here we asked whether the four complexes with different affinities could be distinguished on a ^1^H,^15^N heteronuclear single-quantum coherence (HSQC) spectrum during the phosphorylation reaction. To investigate this, we compared the spectra of ^15^N-labeled p62-PH complexed with unphosphorylated, Ser46-phosphorylated, Thr55-phosphorylated and Ser46, Thr55 double-phosphorylated p53-TAD2 peptides. Notably, many signals from interacting residues and nearby residues of p62-PH, such as E58 and T74, could be distinguished from each other in the four different affinity complexes (Figures 3b and c and Supplementary Figure S4). We therefore considered that, by using real-time NMR to monitor these distinctive signals from p62-PH in the transition from the p53-TAD2-unphosphorylated, low-affinity complex, through the singly phosphorylated, high-affinity complex, to the dual phosphorylated, highest-affinity complex during the phosphorylation reaction, we would be able to follow the increasing interaction between p53-TAD phosphorylated at Ser46/Thr55 and p62-PH. Furthermore, if ^15^N-labeled p53-TAD were used, then the site-specific phosphorylation reaction could be monitored at the same time, as mentioned above (Figure 2).

### Real-time and simultaneous monitoring of the dual phosphorylation of p53-TAD and its increasing interaction with p62-PH

**Figure 4:**
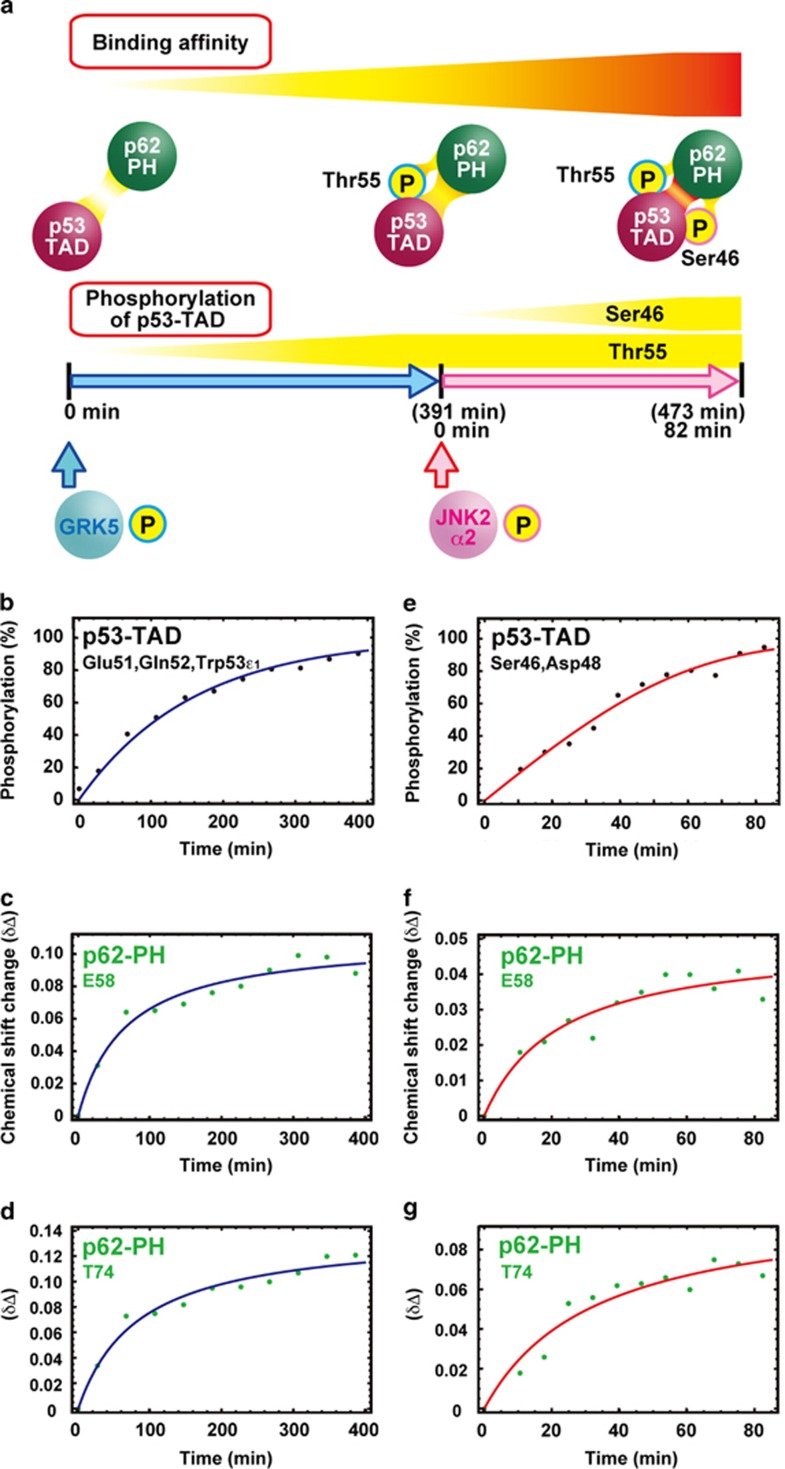
Real-time NMR monitoring of the simultaneous site-specific phosphorylation of p53-TAD and its strengthening interaction with p62-PH. (**a**) Experimental scheme. (**b**) Phosphorylation of p53-TAD at Thr55 by GRK5. (**c** and **d**) Chemical shift changes of the p62-PH residues E58 and T74, respectively, after addition of GRK5. (**e**) Phosphorylation of p53-TAD at Ser46 by JNK2α2. (**f** and **g**) Chemical shift changes of the p62-PH residues E58 and T74, respectively, after addition of JNK2α2.

In order to monitor the interplay between the p53 TAD2 kinases JNK2α2 and GRK5, p53-TAD2 and p62-PH in real time, we designed the experimental procedure shown in Figure 4a. The experiment was started by addition of 2.5 μg of GRK5 into 150 μl of 25 μM solution of a complex of ^15^N-labeled p53-TAD and ^15^N-labeled p62-PH in an NMR tube. Unfortunately, the signal from Thr55 disappeared when p53-TAD bound to p62-PH. However, because phosphorylation of a residue changes the local environment, as reflected in the chemical shift of residues around the phosphorylation site, we were able to follow the phosphorylation reaction of Thr55 by monitoring signals from the neighboring Glu51, Gln52 and Trp53 residues of p53-TAD (Supplementary Figure S5).

Phosphorylation of Thr55 of p53-TAD exponentially increased and reached ~100% at ~390 min (Figure 4b). Alongside the progression of the phosphorylation reaction at Thr55, signals from E58 and T74 of p62-PH started moving from their positions in the low-affinity complex toward their positions in the high-affinity complex immediately after the addition of GRK5 and stopped moving when the phosphorylation of Thr55 of p53-TAD was complete (Figures 4c and d).

The sample was then removed from the NMR magnet, 1.0 μg of JNK2α2 was added at 391 min and NMR monitoring was immediately resumed. Phosphorylation of Ser46 of p53-TAD by JNK2α2 increased exponentially and reached saturation at ~80 min (Figure 4e). The phosphorylation reaction at Ser46 in the Thr55-phosphorylated p53-TAD in complex with p62-PH was relatively slow as compared with p53-TAD alone or when unphosphorylated p53-TAD in complex was used as a substrate (Figure 2b vs Figure 4e and Supplementary Figure S6). Upon addition of JNK2α2, signals from E58 and T74 in p62-PH started shifting from their positions in the high-affinity complex to their positions in the highest-affinity complex (Figures 4f and g). At the same time that the phosphorylation reaction of Ser46 reached saturation, these signals stopped shifting.

### A phosphorylation site in XPC that affects its interaction with p62-PH

Next, we applied the NMR method to a complex of XPC with p62-PH in order to identify a residue in XPC that would affect the intermolecular interaction by its phosphorylation. To this end, we initially identified the p62-PH-binding site in XPC by NMR. No tertiary structures of the complex between human XPC-AF and p62-PH have been determined; however, a complex structure of the AF of Rad4, the budding yeast homologue of human XPC, bound to the PH domain of Tfb1, the yeast homologue of the human TFIIH p62 subunit is available.^21^ Keeping this in mind, we first examined the complex structure of human XPC-AF bound to human p62-PH, while noting that the complex structure of p53-TAD bound to human p62-PH differs considerably from that of p53-TAD bound to yeast Tfb1-PH owing to different surface characteristics of the PH domains of p62 and Tfb1.^18^

**Figure 5:**
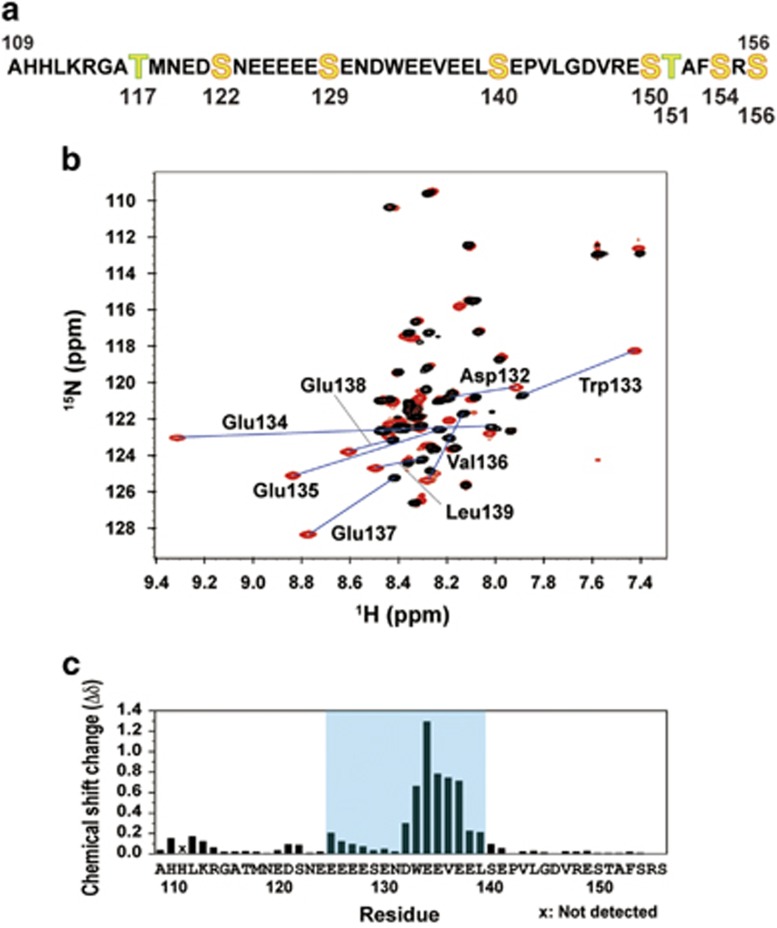
Identification of the binding site for p62-PH in XPC-AF. (**a**) Amino-acid sequence of XPC-AF. (**b**) Overlay of ^1^H,^15^N HSQC spectra of XPC-AF before (black) and after (red) the addition of p62-PH. Residues showing a large chemical shift change are labeled. (**c**) Histogram showing the chemical shift change (*Δδ*) per residue of XPC-AF. The binding site identified for p62-PH is highlighted in cyan.

We prepared ^13^C/^15^N-labeled XPC-AF (residues 109–156) (Figure 5a). Similar to p53-TAD, which contains seven serine and two threonine residues, XPC-AF contains six serine and two threonine residues (Figure 1a) and is likely to be largely unstructured in the unbound state. The addition of p62-PH to XPC-AF drastically dispersed several specific signals of XPC-AF on the ^1^H,^15^N HSQC spectrum, indicating the formation of a stable structure of the complex (Figure 5b). Signal assignment for XPC-AF identified residues 125–139 as the binding site for p62-PH (Figure 5c).

### Phosphorylation of Ser129 in XPC-AF increases its affinity for p62-PH

**Figure 6:**
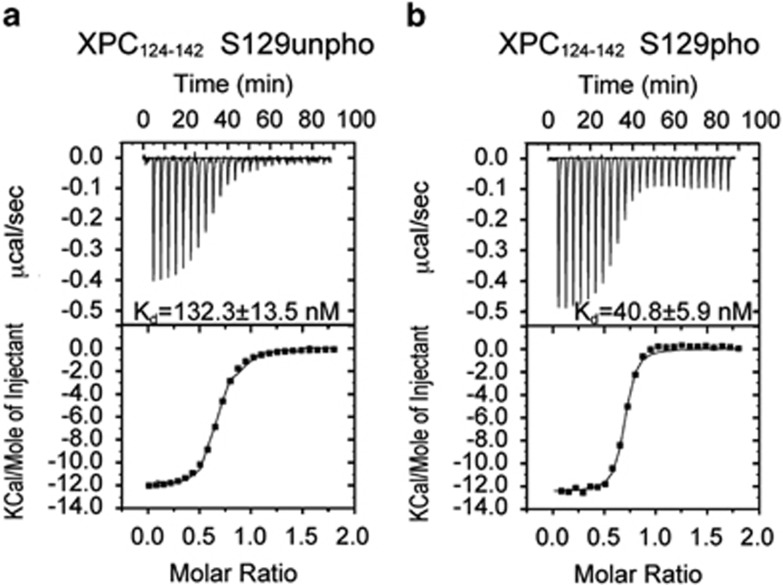
Effect of the phosphorylation of Ser129 in XPC on its interaction with p62-PH. (**a** and **b**) Thermograms (upper panel) and binding isotherms (lower panel) of the calorimetric titration in ITC experiments. Titrant: (**a**) XPC peptide (residue 124–142) unphosphorylated (unpho) at Ser129, and (**b**) XPC peptide (residue 124–142) phosphorylated (pho) at Ser129. The calculated *K*_d_ values (means±s.d.) are indicated in the upper panels.

The binding site in XPC-AF contained one serine residue, Ser129, but it was unknown whether phosphorylation of this residue would have any effect on its interaction with p62-PH. We therefore compared the binding activities of an XPC peptide (residues 124–142) that was unphosphorylated and phosphorylated at Ser129 by using ITC. The unphosphorylated XPC peptide bound to p62-PH with a dissociation constant (*K*_d_) of 132 nM (Figure 6a), whereas the Ser129-phosphorylated XPC peptide showed ~3.2-fold stronger binding to p62-PH with a *K*_d_ of 41 nM (Figure 6b).

### CK2 specifically phosphorylates Ser129 of XPC-AF

**Figure 7:**
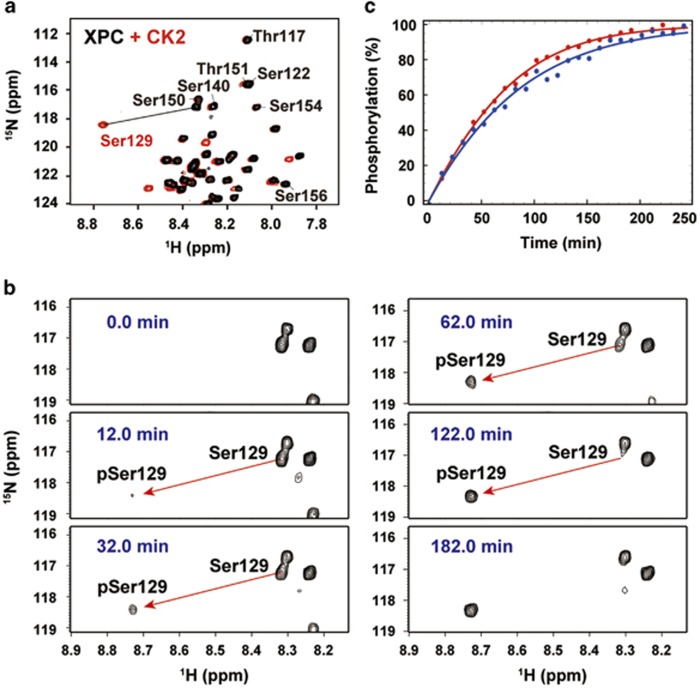
Site-specific phosphorylation of XPC-AF at Ser129 by CK2. (**a**) Overlay of ^1^H,^15^N HSQC spectra of XPC-AF alone (black) and a mixture of XPC-AF and CK2 (red). (**b** and **c**) Real-time NMR monitoring of the phosphorylation of XPC-AF at Ser129 by CK2. (**b**) ^1^H,^15^N HSQC spectra of XPC-AF before and after the addition of CK2. (**c**) Build-up curves showing time course of the phosphorylation of XPC-AF at Ser129 by CK2. The curves are calculated by using data from Ser129 and its neighboring residues (Supplementary Figure S7). Each experiment was repeated twice.

Considering the sequence context of Ser129 in XPC-AF, we reasoned that CK2 would be likely to phosphorylate Ser129, together with Ser122 and Ser140. To examine the activity and specificity of CK2 for XPC-AF, we added 2.5 μg of CK2 to 150 μl of 50 μM ^15^N-labeled XPC-AF and monitored the phosphorylation reaction using NMR. CK2 specifically phosphorylated only Ser129 (Figure 7a). Next, we used real-time NMR to investigate the kinetics of the enzymatic reaction of CK2 and XPC-AF. Using a substrate of 150 μl of 50 μM ^15^N-labeled XPC-AF, 2.5 μg of CK2 achieved ~100% phosphorylation of Ser129 within ~3 h (Figures 7b and c and Supplementary Figure S7).

### Real-time and simultaneous monitoring of the phosphorylation and enhanced interaction of XPC-AF with p62-PH

**Figure 8:**
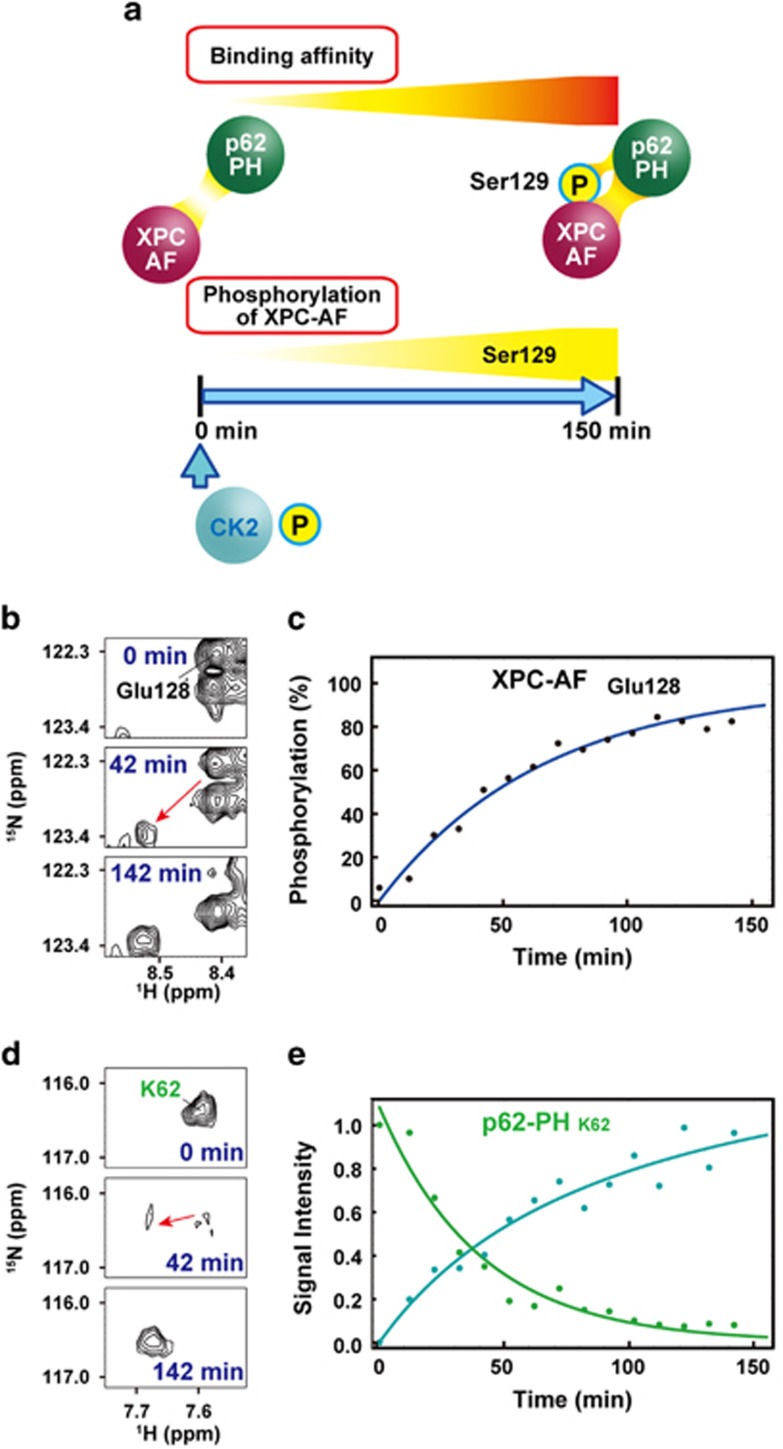
Real-time NMR monitoring of the simultaneous site-specific phosphorylation of XPC-AF and its strengthening interaction with p62-PH. (**a**) Experimental scheme. (**b** and **c**) Real-time NMR monitoring of the phosphorylation of XPC-AF at Ser129 by CK2. (**b**) ^1^H,^15^N HSQC spectra of the complex between XPC-AF and p62-PH before, and 42 and 142 min after the addition of CK2 showing signals from Glu128 of XPC-AF. (**c**) Build-up curve showing time course of the phosphorylation of XPC-AF at Ser129 by CK2. The curve is calculated by using signals from Glu128 of XPC-AF. (**d** and **e**) Real-time NMR monitoring of the increasing interaction between XPC-AF and p62-PH. (**d**) ^1^H,^15^N HSQC spectra of the complex between XPC-AF and p62-PH before, and 42 and 142 min after the addition of CK2 showing signals from K62 of p62-PH. (**e**) Plot of changes in the signal intensity of K62 in p62-PH in complex with unphosphorylated XPC-AF (green) and Ser129-phosphorylated XPC-AF (cyan).

To monitor the process whereby the binding between XPC-AF and p62-PH becomes a high-affinity interaction by the phosphorylation of XPC-AF at Ser129 in real time, we designed the experiment shown in Figure 8a. NMR spectra were collected starting from the addition of 2.5 μg of CK2 to 150 μl of a 50 μM solution of a complex of ^15^N-labeled XPC-AF and ^15^N-labeled p62-PH in an NMR tube. In monitoring the phosphorylation of XPC-AF at Ser129 in the unbound state, we utilized signals from the neighboring residues, Glu128 and Asn131, in addition to Ser129 (Supplementary Figure S7). In the complex, several signals from XPC-AF, including Ser129 and Asn131, overlapped with those from p62-PH; however, we could monitor the phosphorylation of Ser129 by using isolated signals from Glu128 (Figure 8b). Phosphorylation of Ser129 increased exponentially and reached ~80% at ~2 h (Figure 8c). During this time, several limited signals from p62-PH changed. K62 was the residue that was most affected with respect to chemical shift (Supplementary Figure S8). The signal from K62 of p62-PH observed at 0 min gradually disappeared as the phosphorylation of Ser129 of XPC-AF increased (Figure 8d). As the same time, a new signal, corresponding to K62 of p62-PH in complex with Ser129-phosphorylated XPC-AF, gradually appeared. Whereas the intensity of the former signal exponentially decreased (Figure 8e), that of the latter exponentially increased (Figure 8e), similar to the build-up curve of the increasing signal from Glu128 of XPC-AF (Figure 8c).

## Discussion

For studies on multifunctional proteins such as p53, in which each of many phosphorylation sites is intimately associated with a particular function by regulating intermolecular interactions, real-time NMR spectroscopy can be a very useful method for observing not only changing phosphorylation states but also the resulting changes in interactions with the partners. To demonstrate this, here we focused on phosphorylated p53-TAD2 and its interaction with one of its targets, p62-PH of the general transcription factor TFIIH, because of the availability of the structure of this complex.^18^ Knowledge of tertiary structure can help us to correctly interpret experimental results. Although eight tertiary structures of unphosphorylated p53-TAD (TAD1 and/or TAD2) bound to its targets have been determined,^17, 29, 30, 31, 32, 33, 34^ a structure containing phosphorylated p53-TAD has only been solved for p53-TAD2 phosphorylated at Ser46 and Thr55 bound to p62-PH.^18^ Interestingly, in contrast to the amphipathic helix structures of unphosphorylated p53-TAD1 and p53-TAD2 observed in the other complexes, phosphorylated p53-TAD2 in this structure forms an extended string-like structure enabling it to make extensive and optimal contacts (Figure 3a).

Unexpected results were obtained by NMR regarding the specificities of seven enzymes known as p53-TAD2 kinases (Figure 1). Only JNK2α2 for Ser46 and GRK5 for Thr55 were found to be site-specific kinases for p53-TAD. The kinetics of the phosphorylation reaction analyzed by NMR in real time indicated that these kinases are efficient enzymes (Figure 2). It was previously reported that transient overexpression of GRK5 in osteosarcoma cells significantly increased phosphorylation of p53 at Thr55, whereas knockdown of GRK5 decreased it; in addition, Thr55 phosphorylation promoted degradation of p53, leading to inhibition of p53-dependent DNA damage-induced apoptosis.^28^ Phosphorylation of Thr55 of p53 by GRK5 was also observed to occur during the G_2_/M phase of the cell cycle in a normal retinal pigment epithelial cell line, contributing to the regulation of p53 expression.^35^ By contrast, phosphorylation at Ser46 by JNK2α2 *in vivo* has not been reported; as a result, experiments on the transient overexpression of JNK2α2 and knockdown of JNK2α2 in cells remain to be conducted in future studies. However, we do not rule out the possibility that these unexpected results are due to the conditions used in the experiments. Another possibility is interplay among phosphorylation sites; for example, phosphorylation of some residues might require prior phosphorylation at another specific site. It will be interesting to test these possibilities by NMR.

The four different affinity complexes between p53-TAD and p62-PH arising from different phosphorylation states of p53-TAD—namely, the low-affinity complex (unphosphorylated p53-TAD), two moderate-affinity complexes (p53-TAD singly phosphorylated at Ser46 or Thr55) and the high-affinity complex (p53-TAD doubly phosphorylated at Ser46 and Thr55)—could be distinguished from each other via the signals from p62-PH on a ^1^H,^15^N HSQC spectrum (Figure 3). Thus the real-time NMR monitoring system enabled us to successfully observe the process of site-specific phosphorylation of p53-TAD via signals from p53-TAD simultaneously with the formation of complexes of increasing affinity via signals from p62-PH (Figure 4). Quantitative analysis of the ^1^H,^15^N HSQC spectrum for a complex in which both proteins are labeled with ^15^N can incur problems with overlapping signals because of the increasing numbers of residues. In the present method, however, such a problem was overcome by using isolated signals from residues that are influenced by the difference in affinity between complexes, even though they are not directly interacting residues and the difference is small. The contact surfaces on p62-PH are essentially identical in the four complexes.^18^ E58 and T74 of p62-PH, which were utilized to discriminate the complexes, do not directly interact with p53-TAD2 (Figure 3). E58 is located ~17 and ~12 Å away from phosphorylated Ser46 and Thr55, respectively, in p53-TAD2 (backbone N–N distance), while T74 is ~10 and ~14 Å away, respectively. The difference in affinity was found to be approximately fivefold in *K*_d_ between the unphosphorylated complex and the Thr55-phosphorylated complex and between the Thr55-phosphorylated complex and the Ser46, Thr55 double-phosphorylated complex. Considering these factors, the differences in the chemical shift values of E58 and T74 of p62-PH among the four affinity complexes would be rather small. Nevertheless, changes in E58 and T74 could account for the increases in complex affinity that occurred concomitantly with each phosphorylation of Thr55 and phosphorylation of Ser46 (Figures 4c, d, f and g).

In this way, real-time NMR monitoring is extremely useful in evaluating the impact of phosphorylation on protein–protein interactions, as well as enzymatic activity, specificity and kinetics. In the present study, we used ^1^H and ^15^N atoms of the protein backbone to monitor the altering intermolecular interaction, but other atoms will offer different types of structural information. For example, monitoring ^13^C atoms in a protein backbone can enable the observation of changes in the secondary structure of a protein of interest.

Real-time NMR monitoring has also proved to be beneficial in identifying a key residue involved in modulating an interaction with a partner via phosphorylation. Similar to p53-TAD, XPC-AF has many potential phosphorylation sites (Figures 5a and 1a). According to the posttranslational modification database, PHOSIDA,^22, 23^ Thr117, Ser122 and Ser129 in XPC-AF are phosphorylated *in vivo*; to date, however, no studies on the role of these phosphorylated residues have been reported. Notably, the binding site for p62-PH identified in XPC-AF during the NMR chemical shift perturbation experiment contained a serine residue, Ser129 (Figure 5). The small change in the chemical shift of Ser129 in XPC-AF upon binding to p62-PH suggested that unphosphorylated Ser129 is unlikely to directly participate in the interaction (Figure 5). From this result, we speculated that phosphorylated Ser129 might alter the affinity of XPC-AF for p62-PH. An ITC experiment demonstrated that phosphorylation at Ser129 increases the binding affinity ~3.2-fold (Figure 6).

To observe the increasing affinity of XPC-AF for p62-PH due to phosphorylation of Ser129 in real time, we needed to identify a candidate kinase. Real-time NMR spectroscopy revealed that CK2, which was predicted to be a likely kinase from the sequence context, specifically and efficiently phosphorylated XPC-AF at Ser129, despite the presence of two other potential phosphorylation sites (Figure 7). Thus, by using real-time NMR monitoring, we could observe the transition from the high-affinity complex to the higher-affinity complex resulting from the CK2-mediated phosphorylation of Ser129 in XPC-AF (Figure 8). In the case of the p53-TAD and p62-PH complex, the signals from p62-PH in the lower-affinity complex gradually moved toward their positions in the higher-affinity complex during the phosphorylation (for example, see E58 and T74 of p62-PH; Figures 4c, d, f and g). In the XPC-AF and p62-PH complex, by contrast, the intensity of signals from p62-PH in the unphosphorylated, lower-affinity complex decreased, while new signals appeared at a different position in the phosphorylated, higher-affinity complex and gradually grew in intensity, consistent with the progression of phosphorylation (for example, see K62 of p62-PH; Figure 8e). This difference is attributed to variations in the exchange rates of the complexes on the NMR timescale: the p53-TAD and p62-PH complexes are in fast exchange (that is, soft complexes), whereas the XPC-AF and p62-PH complexes are in slow exchange (that is, hard complexes). Thus the hardness of a complex can be seen in the changing patterns of the NMR signals of that complex.

To interpret the results of the increased affinity of the XPC-AF and p62-PH complex arising from phosphorylation of Ser129 in XPC-AF from the point of view of tertiary structure, we built a structural model of the complex based on the structure of p53-TAD2 and p62-PH using the Modeller program^36^ (Supplementary Figure S9). In the model, Ser129 of XPC-AF is in close proximity to K62 of p62-PH. Although the correct answer awaits experimental determination of the complex structure, we speculate that the observed increase in binding affinity is due to the generation of an electrostatic interaction between the negatively charged phosphorylated Ser129 of XPC-AF and K62 of p62-PH.

In summary, we have established a real-time NMR system for monitoring the site-specific phosphorylation of p53 and XPC simultaneously with the enhancement in affinity for their target, p62-PH. We have also demonstrated that real-time NMR monitoring can be a valuable tool for exploring phosphorylation-mediated regulatory mechanisms of intermolecular interactions. The present method is not limited to phosphorylation (or dephosphorylation). Other posttranslational modifications such as acetylation and methylation perturb the local environment around the modified site,^37, 38, 39^ thereby altering the chemical shift values of the modified residue and its neighboring residues. It will therefore be possible to investigate the effects of these modifications on protein–target biomolecule interactions, as well as being able to study protein–drug interactions. In addition, in-cell NMR spectroscopy has been extensively developed to characterize the dynamic behavior of proteins in cells.^40, 41, 42, 43, 44, 45, 46^ We expect that the present method will be valuable for *in vivo* studies.

## Materials and methods

### Preparation of human p53-TAD

The ^13^C/^15^N-labeled human p53-TAD (residues 1–73) was prepared as previously described.^18^

### Preparation of human TFIIH p62-PH

The ^13^C/^15^N-labeled human TFIIH p62-PH domain (residues 1–108) was prepared as previously described.^47^

### Preparation of human XPC-AF

The ^13^C/^15^N-labeled human XPC-AF (residues 109–156) was expressed as a hexa-histidine-tagged product in a pET15b vector (Novagen Merck Millipore, Tokyo, Japan) in *Escherichia coli* Rosetta 2 (DE3) pLysS (Novagen Merck, KGaA, Darmstadt, Germany). The cells were grown at 37 °C in M9 minimal medium containing [^15^N]ammonium chloride and [^13^C]glucose. The products were expressed by induction with 1 mM isopropyl-β-d-thiogalactopyranoside. After 20–22 h of growth, the cells were harvested, and the cell pellet was suspended in buffer A (20 mM Tris–HCl (pH 8.0), 10% glycerol, 1m NaCl). The cells were lysed by sonication and centrifuged. The supernatant was loaded onto a Ni-nitrilotriacetic acid–agarose (Qiagen, Tokyo, Japan) column, equilibrated with buffer A. The sample was washed with buffer A containing 20 mM imidazole-HCl and eluted by 500 mM imidazole-HCl. Peak fractions were pooled, and the buffer was changed to thrombin cleavage buffer (10 mM Na_2_HPO_4_, 1.8 mM KH_2_PO_4_, 500 mM NaCl, 2.7 mM KCl, pH 7.3) using Amicon Ultra devices (Merck Millipore, Tokyo, Japan). The sample was digested with thrombin for 13–20 h at 25 °C to remove the histidine tag. After concentration using Amicon Ultra, the sample was applied to a Superdex30 (GE Healthcare, Tokyo, Japan) column equilibrated with 20 mM potassium phosphate (pH 6.8) and 550 mM NaCl.

### Preparation of peptides

Unphosphorylated, Ser46- and Thr55-phosphorylated and Ser46, Thr55 double-phosphorylated peptides of human p53-TAD2 (residues 41–62), and unphosphorylated and Ser129-phosphorylated peptides of human XPC (residues 124–142) were purchased from Sigma Genosys Japan (Ishikari, Japan).

### Phosphorylation reaction

CDK5, PKCδ, ERK2, GRK5 and JNK2α2 were purchased from Upstate Merck Millipore (Tokyo, Japan) and CK1 and CK2 were purchased from New England Biolabs (Ipswich, MA, USA). For p53, 2.5 μg of each kinase was added to 100 μl of 50 μM ^13^C/^15^N-labeled human p53-TAD. For XPC, 2.5 μg of CK2 was added to 150 μl of 50 μM ^13^C/^15^N-labeled human XPC-AF. Phosphorylation was conducted in 20 mM potassium phosphate (pH 6.8), 1 mM ATP, 10 mM MgCl_2_ and 10% D_2_O and monitored at 25 °C on a Bruker AVANCE III HD 600 MHz spectrometer (Bruker, Rheinstetten, Germany) equipped with a triple-resonance TCI cryogenic probe. The ^1^H,^15^N HSQC spectra shown in Figures 1c–i were taken at 1 day (CDK5), 1 day (PKCδ), 17 h (CK1), 9 h (ERK2), 1 day (CK2), 1 h (JNK2α2) and 21 h (GRK5) after addition of the respective kinase. The ^1^H,^15^N HSQC spectrum shown in Figure 7a were taken at 1 h after the addition of CK2. Spectra were processed by NMRPipe^48^ and analyzed by NMRView.^49^

### Real-time NMR monitoring of phosphorylation

Real-time NMR experiments and calculation of build-up curves of the phosphorylation reactions have been described by Selenko *et al.*^24^ For p53, 1.0 μg of JNK2α2 and 2.5 μg of GRK5 were added to 150 μl of 25 μM ^13^C/^15^N-labeled human p53-TAD alone and in complex with ^13^C/^15^N p62-PH in 20 mM potassium phosphate (pH 6.8), 1 mM ATP, 10 mM MgCl_2_, 5 mM deuterated dithiothreitol (DTT) and 10% D_2_O. For XPC, 2.5 μg of CK2 was added to 150 μl of 50 μM ^13^C/^15^N-labeled XPC-AF alone and in complex with ^13^C/^15^N-labeled p62-PH in 20 mM potassium phosphate (pH 6.8), 1 mM ATP, 10 mM MgCl_2_, 5 mM deuterated DTT and 10% D_2_O. The phosphorylation of p53 TAD at Ser46 by JNK2α2 and at Thr55 by GRK5 was monitored by ^1^H-^15^N SOFAST-HMQC^50^ and ^1^H-^15^N fast HSQC,^51^ respectively. The phosphorylation of XPC-AF at Ser129 by CK2 was monitored by ^1^H-^15^N fast HSQC. Each experiment was repeated twice. Spectra were processed by NMRPipe and analyzed by NMRView. Build-up curves showing the time course of phosphorylation of p53-TAD at Ser46 by JNK2α2 were calculated by using data from Leu45, Ser46, Asp48, Ile50 and Gln52 (Supplementary Figures S3a–c). Those of phosphorylation of p53-TAD at Thr55 by GRK5 were calculated by using data from Glu51, Gln52, Trp53, Thr55 and Glu56 (Supplementary Figures S3d–f). Those of the phosphorylation of XPC-AF at Ser129 by CK2 were calculated by using data from Glu128, Ser129 and Asn131 (Supplementary Figure S7).

### NMR perturbation

Unphosphorylated, Ser46- and Thr55-phosphorylated and Ser46, Thr55 double-phosphorylated peptides of human p53-TAD2 were added to ^15^N-labeled p62-PH at a molar ratio of 1:2.5 (p62:p53) in 20 mM potassium phosphate (pH 6.8), 1 mM ATP, 10 mM MgCl_2_, 5 mM deuterated DTT and 10% D_2_O. Unlabeled p62-PH was added to ^15^N-labeled XPC-AF at a molar ratio of 1:1.2 (XPC:p62) in 20 mM potassium phosphate (pH 6.8), 5 mM deuterated DTT and 10% D_2_O. ^1^H,^15^N HSQC spectra were taken before and after the addition of unlabeled samples at 32 °C on a Bruker AVANCE III HD 600 MHz spectrometer equipped with a triple-resonance TCI cryogenic probe. Backbone resonances of p62-PH and XPC-AF in their unbound and bound forms were assigned with a combination of CBCA(CO)NH, CBCANH, HNCO and HN(CA)CO.^52^ Spectra were processed by NMRPipe and analyzed by NMRView. Chemical shift change (*Δδ*) was calculated as *Δδ*={(*Δδ*^1^H)^2^+(*Δδ*^15^N/5)^2^}^1/2^.

### Isothermal titration calorimetry

The *K*_d_ values of p62-PH and XPC peptide (residues 124–142) were measured by ITC using a VP-ITC calorimeter (Microcal, Northampton, MA, USA). Calorimetric titrations between 100 μM XPC in the syringe (25 × 20 μl injections) and 2 ml of 10 μM p62-PH in the cell were carried out at 20 °C in 20 mM potassium phosphate (pH 6.8). Each injection took place in 4 s with a preinjection delay of 210 s and a syringe stirring speed of 307 r.p.m. Data were analyzed using the Origin software package (MicroCal). *K*_d_ values were presented as means±s.d.
