# Supplementary material for: Real-time and simultaneous monitoring of the phosphorylation and enhanced interaction of p53 and XPC acidic domains with the TFIIH p62 subunit
Source: Oncogenesis. 2015 Jun 1;4(6):e150–. doi: 10.1038/oncsis.2015.13 (PMC4753521; doi:10.1038/oncsis.2015.13)
Supplement: Supplementary Information [file oncsis201513x1.pdf]

Real-time and simultaneous monitoring of the phosphorylation and enhanced interaction of p53 and XPC acidic domains with the TFIIH p62 subunit

Masahiko Okuda[1] and Yoshifumi Nishimura[1]

[1]Graduate School of Medical Life Science, Yokohama City University, Yokohama, Japan.

Correspondence: Professor Y Nishimura, Graduate School of Medical Life Science, Yokohama City University, 1-7-29 Suehiro-cho, Tsurumi-ku, Yokohama 230-0045, Japan

E-mail: nisimura@tsurumi.yokohama-cu.ac.jp

Tel.: +81-45-508-7211

Fax: +81-45-508-7360

SUPPLEMENTARY INFORMATION

Figure S1. Verification of the enzymatic activity of CDK5 and PKCδ. (**a**) The CDK5 substrate peptide, histone H1. A Tyr residue is added to the C-terminus for concentration determination using UV absorption. (**b**) ^{1}H,^{15}N-HSQC spectrum of 10.0 mM histone H1 peptide. (**c**) ^{1}H,^{15}N-HSQC spectrum of 2.0 mM histone H1 peptide. (**d**) ^{1}H,^{15}N-HSQC spectrum of 2.0 mM histone H1 peptide phosphorylated by adding 2.5 μg of CDK5. (**e**) Superposition of the ^{1}H,^{15}N-HSQC spectra in (**c**) and (**d**). (**f**) The PKCδ substrate peptide, PKCtide. A Tyr residue is added to the N-terminus. (**g**) ^{1}H,^{15}N-HSQC spectrum of 3.4 mM PKCtide peptide. (**h**) ^{1}H,^{15}N-HSQC spectrum of 2.0 mM PKCtide peptide. (**i**) ^{1}H,^{15}N-HSQC spectrum of 2.0 mM PKCtide peptide phosphorylated by adding 2.5 μg of PKCδ. (**j**) Superposition of the ^{1}H,^{15}N-HSQC spectra in (**h**) and (**i**). pThr: phosphorylated threonine, pSer: phosphorylated serine. Buffer solution: 20 mM potassium phosphate (pH 6.8), 10% D_2O (**b** and **g**); 20 mM potassium phosphate (pH 6.8), 10 mM ATP, 5 mM $MgCl_2$, 10% D_2O (**c** and **d**); and 20 mM potassium phosphate (pH6.8), 4 mM ATP, 5 mM $MgCl_2$, 10% D_2O (**h** and **i**). The ^{1}H,^{15}N-HSQC experiments were performed by using the

natural abundance of the ^{15}N isotope at 25 °C (**b** and **g**) or 32 °C (**c**, **d**, **h** and **i**) on a Bruker AVANCE III HD 600 MHz spectrometer equipped with a triple-resonance TCI cryogenic probe.

Figure S2. Real-time NMR monitoring of the phosphorylation of p53-TAD at Ser46 and Thr55 on a $^{1}H,^{15}N$-HSQC spectrum. (**a**–**e**) Phosphorylation of p53-TAD at Ser46 by JNK2α2. (**f**–**j**) Phosphorylation of p53-TAD at Thr55 by GRK5.

Figure S3. Build-up curves showing the time course of phosphorylation of p53-TAD at Ser46 and Thr55. Shown are the build-up curves for (**a**) Ser46 phosphorylation, (**b**) the neighboring residues affected by Ser46 phosphorylation, (**c**) Ser46 and the affected residues considered together, (**d**) Thr55 phosphorylation, (**e**) the neighboring residues affected by Thr55 phosphorylation, (**f**) Thr55 and the affected residues considered together. Left panel, 1st experiment; Right panel, 2nd experiment.

Figure S4. ^{1}H,^{15}N-HSQC spectra of ^{15}N-labeled p62-PH in complex with the p53-TAD2 peptide. (**a**) p62-PH alone. (**b**) p62-PH in complex with unlabeled, unphosphorylated p53-TAD2. (**c**) p62-PH in complex with unlabeled, Ser46-phosphorylated p53-TAD2. (**d**) p62-PH in complex with unlabeled, Thr55-phosphorylated p53-TAD2. (**e**) p62-PH in complex with unlabeled, Ser46,Thr55 double-phosphorylated p53-TAD2. (**f**) Superposition of all spectra.

Figure S5. Real-time NMR monitoring of site-specific phosphorylation of the p53-TAD complexed with p62-PH. (**a**) Experimental scheme. (**b**−**e**) Phosphorylation of p53-TAD at Thr55 by GRK5. (**f**−**h**) Phosphorylation of p53-TAD at Ser46 by JNK2α2. The Thr55-phosphorylation reaction was observed by using signals from the backbone amide of Glu51 and Gln52, and the side-chain ε1 of Trp53, because the signal from Thr55 disappeared in the complex with p62-PH.

Figure S6. Comparison of the phosphorylation reaction of p53-TAD at Ser46 by JNK2α2

in different states of p53-TAD. (**a**) p53-TAD alone, (**b**) Unphosphorylated p53-TAD in complex with p62-PH, (**c**) Thr55-phosphorylated p53-TAD in complex with p62-PH.

Figure S7. Build-up curves showing the time course of the phosphorylation of XPC-AF at Ser129. Buildup curves of (**a**) Ser129-phosphorylation, (**b**) the neighboring residues affected by Ser129-phosphorylation, (**c**) Ser129 and the affected residues considered together. Left panel, 1st experiment; right panel; 2nd experiment.

Figure S8. Differences in chemical shift values of ^{15}N labeled p62-PH in complex with unphosphorylated XPC-AF and with Ser129-phosphorylated XPC-AF.

Figure S9. Model of the structure of the complex between XPC-AF and p62-PH.

The structure was built by the MODELLR 9.14 program using the structure of complex of p53-TAD2 and p62-PH (PDB code 2RUK) as a template.

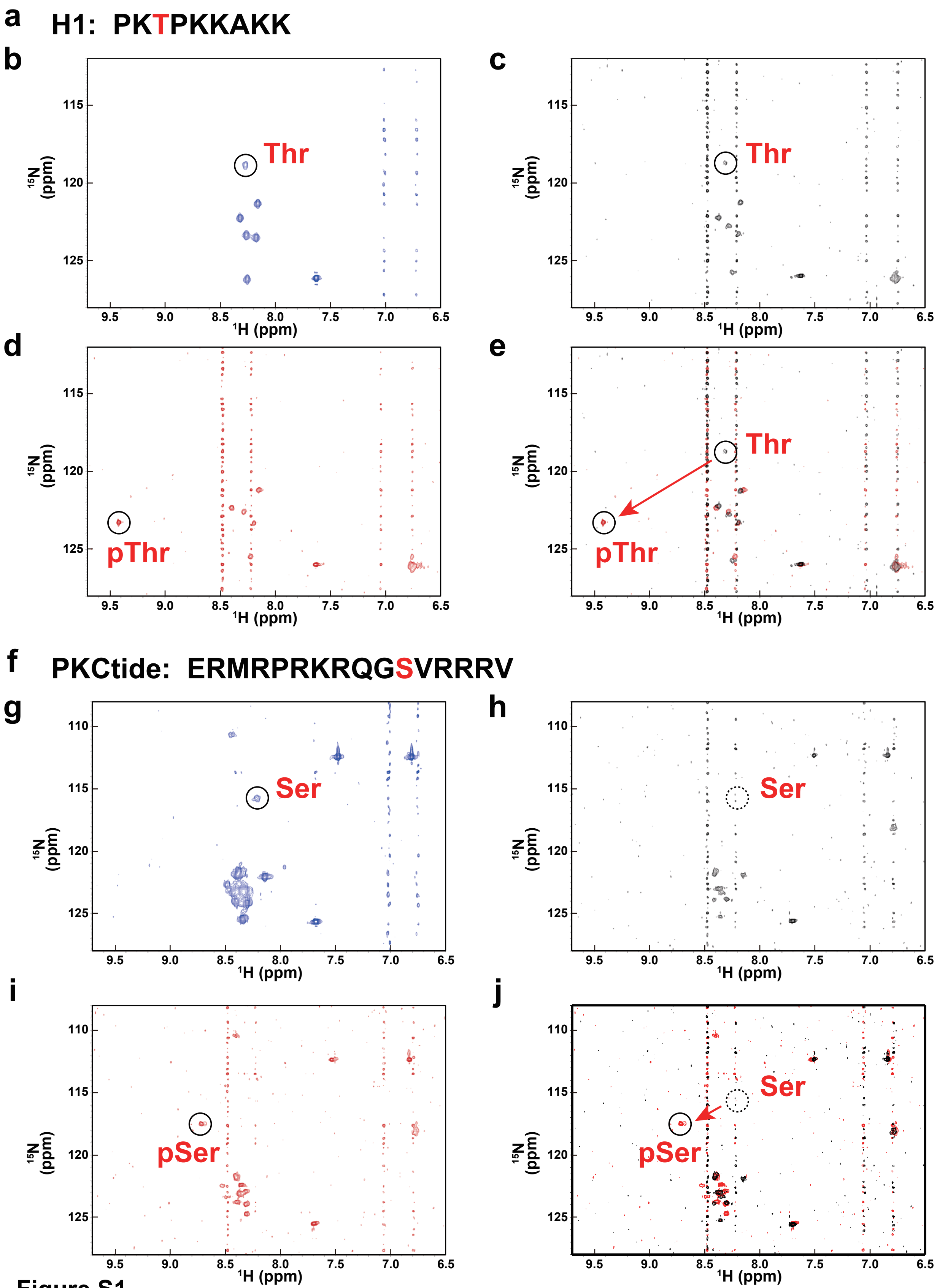
a
H1: PKTPKKAKK
b
c
d
e
Thr
pThr
f
PKCtide: ERMRPRKRQGSVRRRV
g
h
i
j
Ser
pSer
15N (ppm)
1H (ppm)
9.5
9.0
8.5
8.0
7.5
7.0
6.5
110
115
120
125

Figure S1

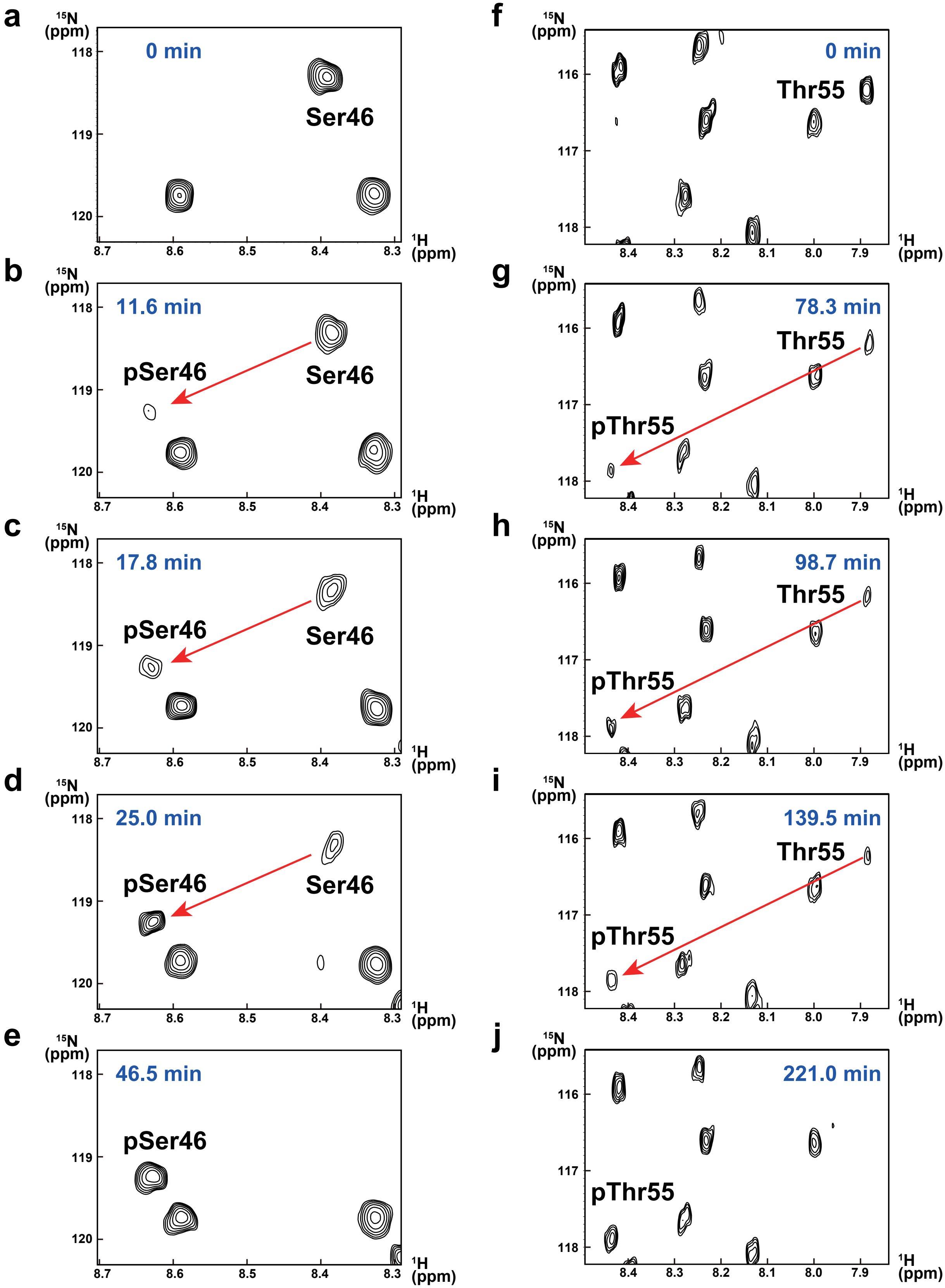
a
b
c
d
e
f
g
h
i
j
0 min
11.6 min
17.8 min
25.0 min
46.5 min
78.3 min
98.7 min
139.5 min
221.0 min
Ser46
pSer46
Thr55
pThr55
15N (ppm)
1H (ppm)

Figure S2

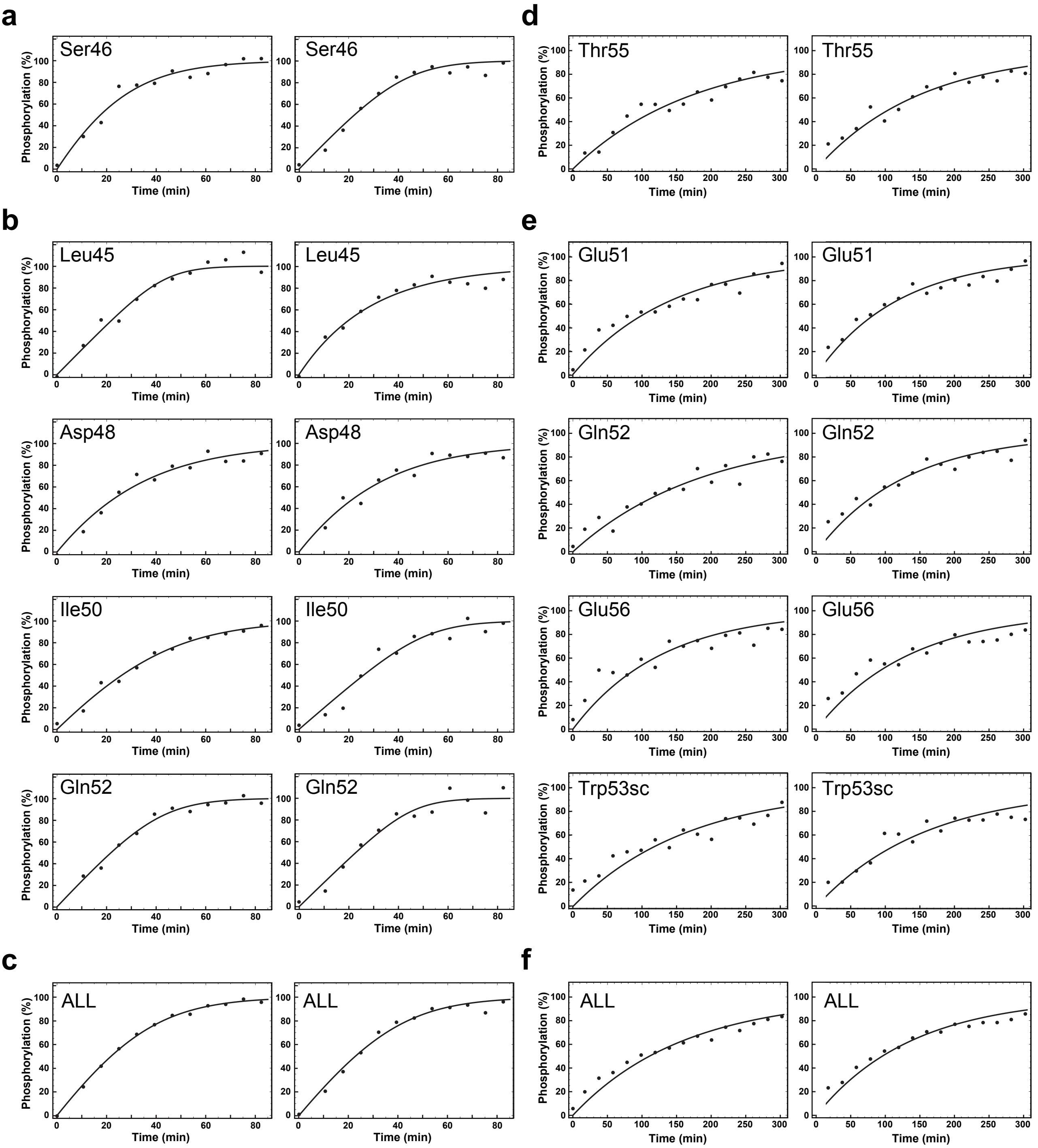
a
Ser46
Ser46
b
Leu45
Leu45
Asp48
Asp48
Ile50
Ile50
Gln52
Gln52
c
ALL
ALL
d
Thr55
Thr55
e
Glu51
Glu51
Gln52
Gln52
Glu56
Glu56
Trp53sc
Trp53sc
f
ALL
ALL
Phosphorylation (%)
Time (min)

Figure S3

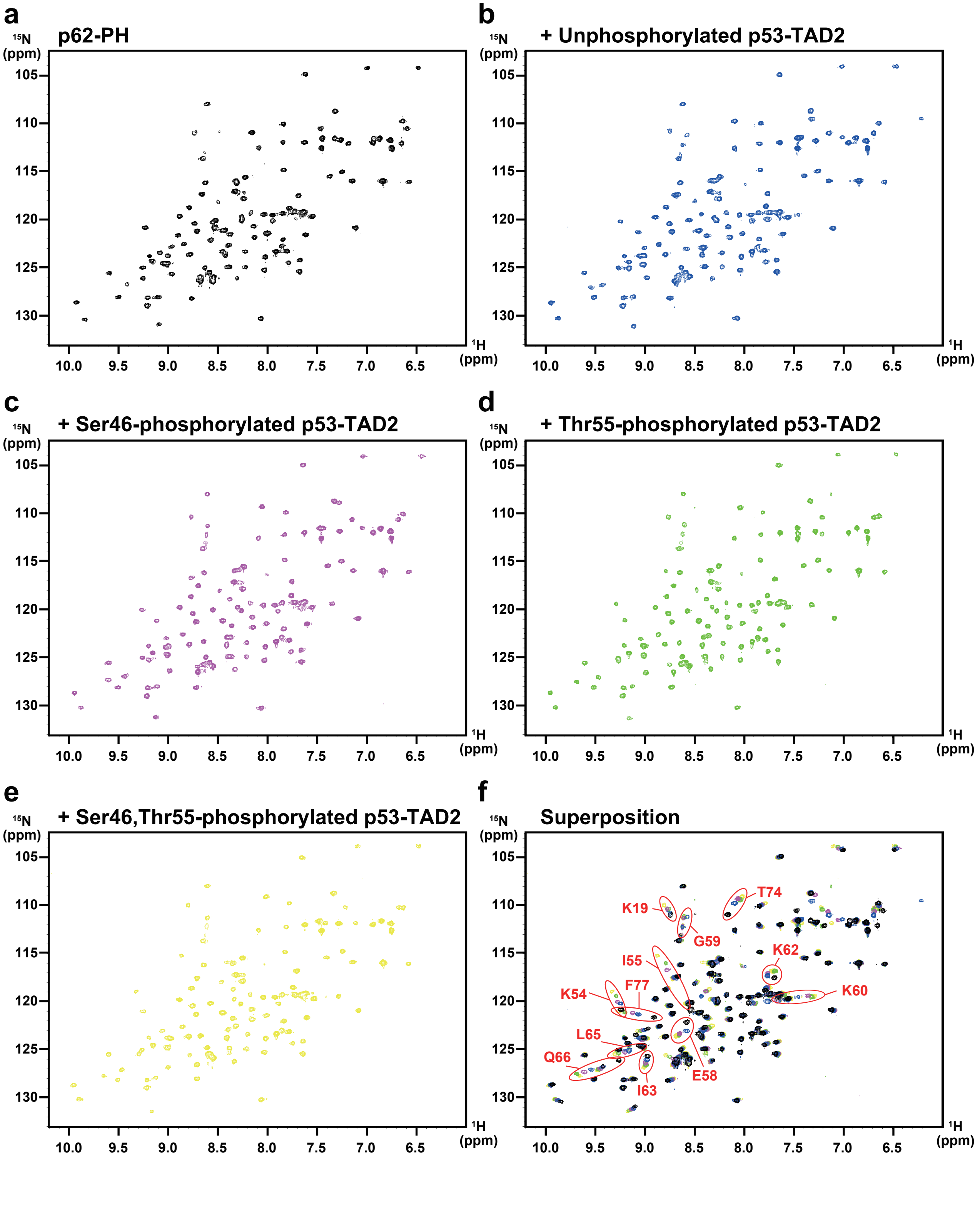
a
p62-PH
b
+ Unphosphorylated p53-TAD2
c
+ Ser46-phosphorylated p53-TAD2
d
+ Thr55-phosphorylated p53-TAD2
e
+ Ser46,Thr55-phosphorylated p53-TAD2
f
Superposition
15N (ppm)
1H (ppm)
105
110
115
120
125
130
10.0
9.5
9.0
8.5
8.0
7.5
7.0
6.5
K19
T74
G59
I55
K62
F77
K54
K60
L65
Q66
I63
E58

Figure S4

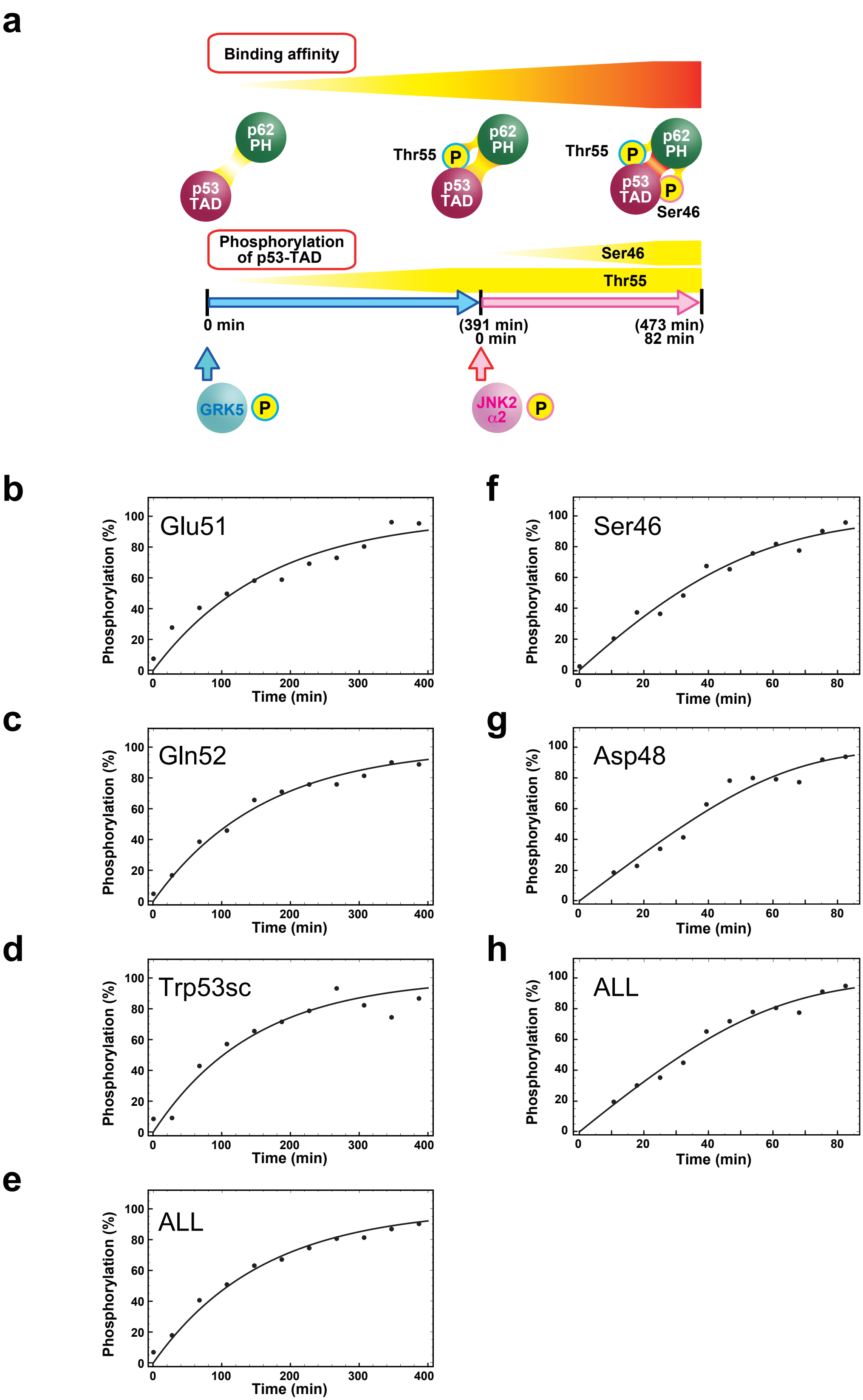

a
Binding affinity
p62 PH
p53 TAD
Thr55
P
Ser46
Phosphorylation of p53-TAD
Ser46
Thr55
0 min
(391 min)
0 min
(473 min)
82 min
GRK5
JNK2 α2
b
Glu51
c
Gln52
d
Trp53sc
e
ALL
f
Ser46
g
Asp48
h
ALL
Phosphorylation (%)
Time (min)

Figure S5

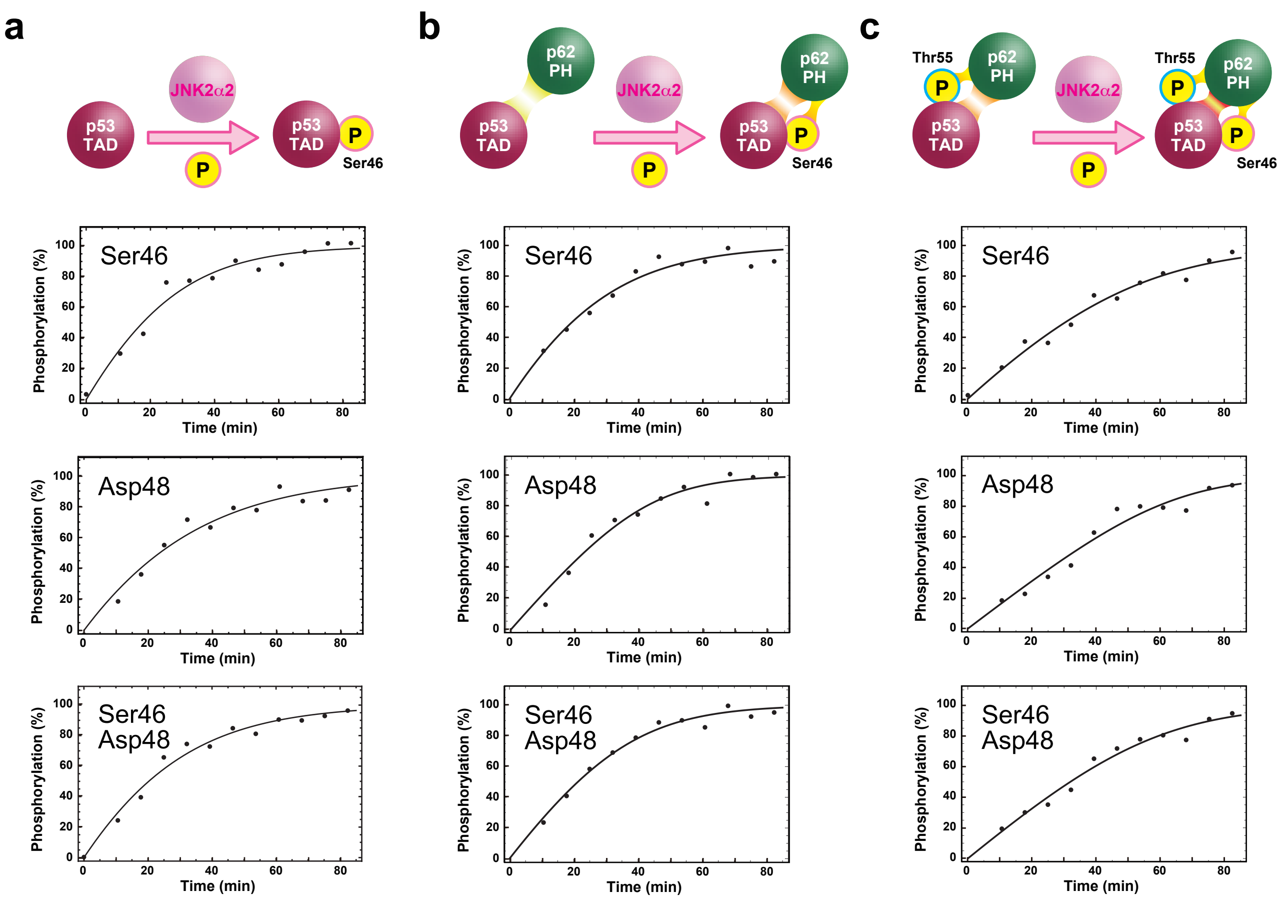
a
b
c
p53 TAD
JNK2α2
P
Ser46
p62 PH
Thr55
Ser46
Asp48
Phosphorylation (%)
Time (min)

Figure S6

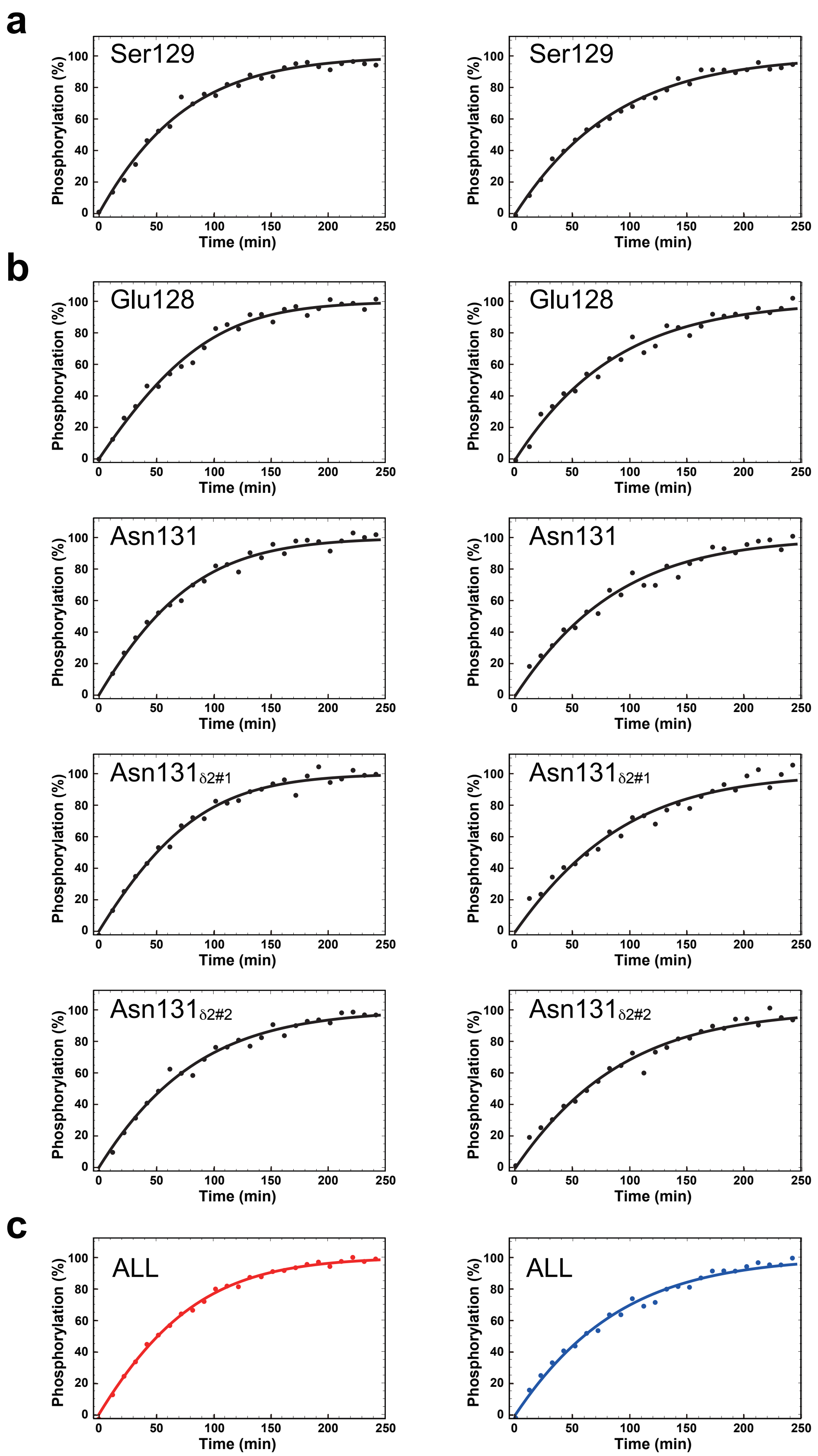
a
b
c
Ser129
Glu128
Asn131
Asn131δ2#1
Asn131δ2#2
ALL
Phosphorylation (%)
Time (min)
0
20
40
60
80
100
0
50
100
150
200
250

Figure S7

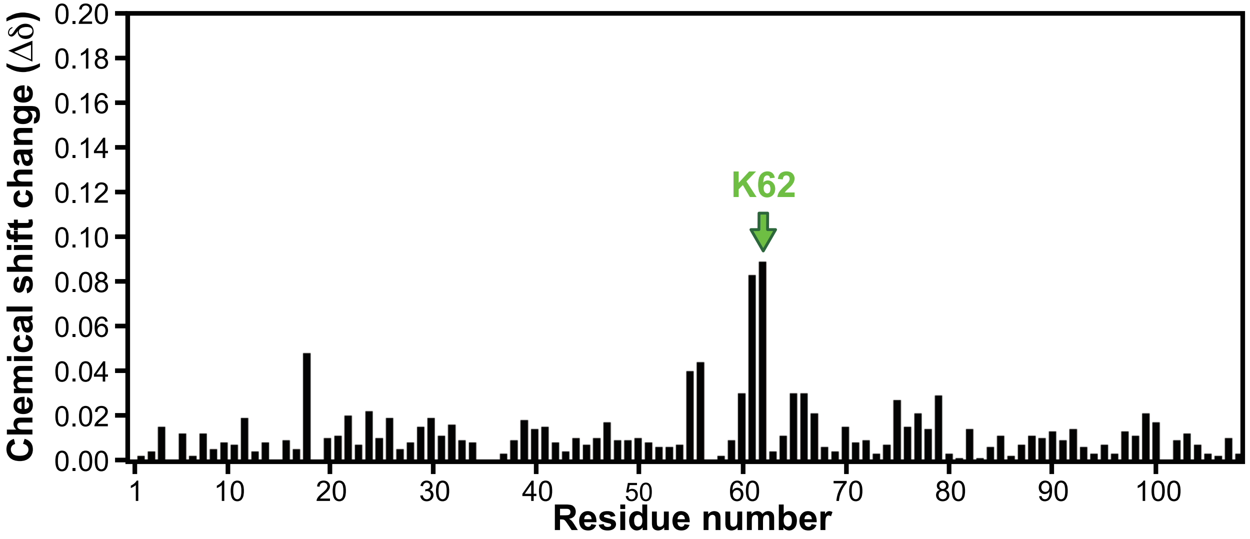

Chemical shift change (Δδ)
0.20
0.18
0.16
0.14
0.12
0.10
0.08
0.06
0.04
0.02
0.00
K62
1
10
20
30
40
50
60
70
80
90
100
Residue number

Figure S8

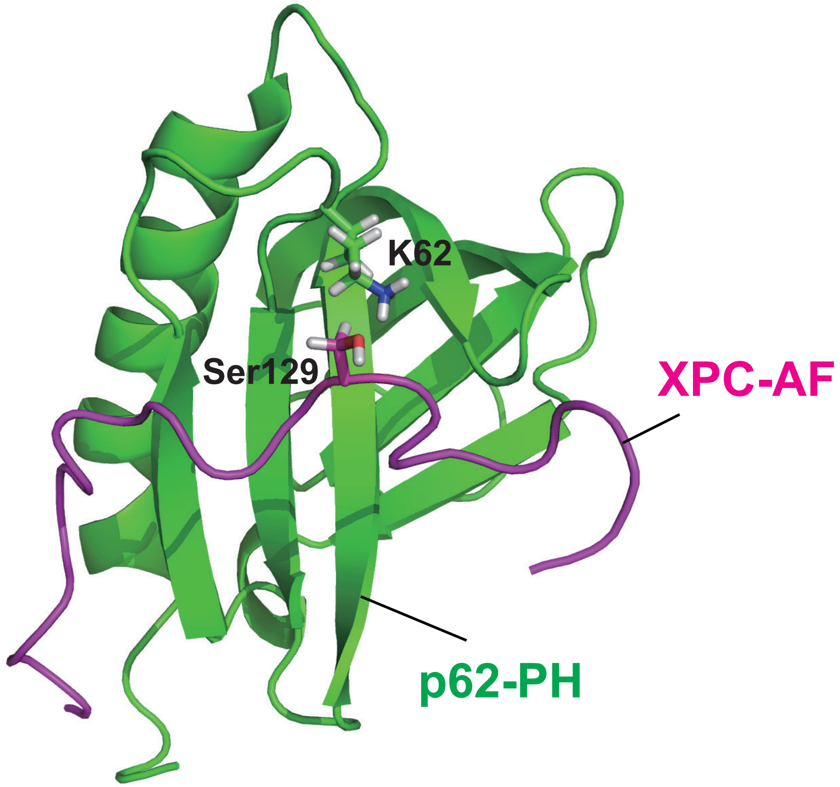

Figure S9
